# Natural Compounds as New Cancer Treatments

**DOI:** 10.3390/medicines6030078

**Published:** 2019-07-23

**Authors:** Enrique Barrajón-Catalán

**Affiliations:** Instituto de Biología Molecular y Celular (IBMC) and Instituto de Investigación, Desarrollo e Innovación en Biotecnología Sanitaria de Elche (IDiBE), Universitas Miguel Hernández (UMH), 03202 Elche, Spain; e.barrajon@umh.es

Cancer is still a global challenge worldwide with a high impact not only on human health, causing morbidity and mortality, but also on economics. Although significant advances have been obtained in early diagnosis and in the development of new drugs, there is still a need of new molecules that contribute to span and improve the actual therapeutic strategies.

Natural compounds from animal, microbial, vegetal, or fungi origin represent countless sources of new compounds that can be used as anticancer drugs if their activity, bioavailability, and toxicity are adequate. There are some general [1] and specific [2] reviews covering this topic, however, the use of natural compounds is not always supported by scientific evidence. This special issue tries to solve this problem, incorporating original manuscripts with new evidence on the use of natural compounds in cancer research and covering the actual state of the art with several reviews.

In this special issue original research manuscripts including both pure compounds and whole natural extracts have been included. Chiang et al. [3] have used sulforaphane, natural molecules previously characterized as promising for fighting cancer [4], to challenge melanoma in combination with the epigenetic drug 5-aza-2′-deoxycytidine (DAC). In this manuscript, the authors have observed that the combination between DAC and sulforaphane reduces melanoma cell growth by changing gene expression profiles but without altering epigenetics. This manuscript is relevant as it demonstrates that the ingestion of some natural compounds can improve the outcome of some cancer treatments. In this sense, this relationship between diet and drugs is not new [5]. There is a long list of interactions between foods and drugs, but these results must be taken in mind carefully as the interactions will not always be beneficious and every case must be studied individually and in the context of a complete and balanced diet. In addition, studies about natural compounds’ bioavailability must also be done to ensure that the dose of the natural compounds that can influence drug treatments is achieved.

This special issue also contains data on the use of whole extracts and complex mixtures. Araki et al. [6] present their results about the use of royal jelly from honey bees on a randomized, double-blind, placebo-controlled trial. Royal jelly is a well-known functional food with relevant health-promoting properties [7]. In their manuscript, Araki et al. focus on patients with renal cell carcinoma under tyrosine kinase inhibitors treatment and observed that those who were supplemented with royal jelly presented lower side effect levels in terms of fatigue and anorexia. This clinical trial does not pretend to introduce royal jelly as an anticancer treatment, but suggests its use as a complementary treatment that could be useful to treat side effects of tyrosine kinase inhibitors treatment. However, further studies increasing the obtained evidence would be interesting before recommending its use in cancer patients, especially those focused on the putative interference between the compounds and the pharmacological treatments.

The last original manuscript of this special issue also studies a complex mixture of natural compounds. Le et al. studied the anticancer potential of a whole natural extract obtained from *Momordica cochinensis* seeds [8]. They performed different extraction procedures and studied the correlation between main compound families and antioxidant, and anticancer activities using different statistical approaches. This is a preliminary study that can be a first step for the future use of these extracts for melanoma treatment research, but before that, a whole extract characterization must be done in order to ensure which compounds are present qualitatively and quantitatively. In addition, expanding the research to other cancer types would be also recommendable.

Reviews are also included in this special issue covering different topics with significant relevance regarding cancer research. Two of these reviews are focused on the pure compound resveratrol. Resveratrol is a stilbene with a long list of biological activities [9], including cardiovascular disease prevention [10], UV protection [11], antiobesity [12], and anticancer properties [13,14]. The first review about resveratrol resumes the actual evidence on the epigenetic changes induced by resveratrol in the context of cancer [15]. It underlines the evidence about the epigenetic action of resveratrol on different types of cancer, including breast, lung, cervical, colon, leukemia, lymphoma, and prostate cancers, providing interesting comments about the existing evidence on each case. The second review about resveratrol is more specific, as it focuses on the role of resveratrol in the expression of the miR-663 microRNA and the relationships between this expression, inflammation, and cancer [16].

Green tea polyphenols have been also reviewed in this special issue. Miyata et al. [17] present in their review an extensive study of the anticancer effects of these polyphenols against bladder cancer. They cover not only in vitro studies using bladder cancer cell lines and in vivo studies using animal models but also new treatment strategies for patients with bladder cancer, based on green tea consumption.

Probiotics are also included in this special issue. Hendler et al. [18] update the previously published evidence on the relationship between probiotics and colon cancer [19,20]. They have written their review with a special interest on animal and human studies. They conclude that the use of probiotics can be recommended to reduce the colon cancer risk by influencing the microbiome composition. However, a more ambitious study, like a metanalysis must be done to reinforce this conclusion.

A global perspective on the use of natural compounds in cancer treatment is taken by Herranz et al. in their review [21]. In this manuscript, the authors provide an interesting point of view about the relevance of multitargeting and synergy when studying natural compounds. The molecular promiscuity of natural compounds has also been discussed by the same authors [22], but in this manuscript they focus on cancer, providing advice on experiment design and presenting the advantages and drawbacks when using complex mixtures of natural compounds as extracts. This last point is especially relevant, as there are still some points that must be improved in natural compounds research if we aspire to be considered as seriously as other disciplines are. As mentioned in this review, correct identification of the compounds, extract reproducibility, and bioavailability studies are the main but not the only weal points of natural product research. Better designs for in vivo trials, the use of non-tumor cell lines as controls of in vitro experiments, and synergy studies with other natural and non-natural compounds, especially clinically used drugs, will be welcome.

Finally, this special issue has met its original objective, contributing to increase the scientific evidence on the use of natural compounds in cancer research and treatment. However, there is a long way ahead, and new studies and evidence must be obtained in the future. Nature is a limitless source of new drugs, but these new drugs must always be supported by strong scientific evidence.
